# Supplementary figures and images for: PhysioFormer: Integrating multimodal physiological signals and symbolic regression for explainable affective state prediction
Source: PLoS One. 2025 Oct 31;20(10):e0335221. doi: 10.1371/journal.pone.0335221 (PMC12578355; doi:10.1371/journal.pone.0335221)

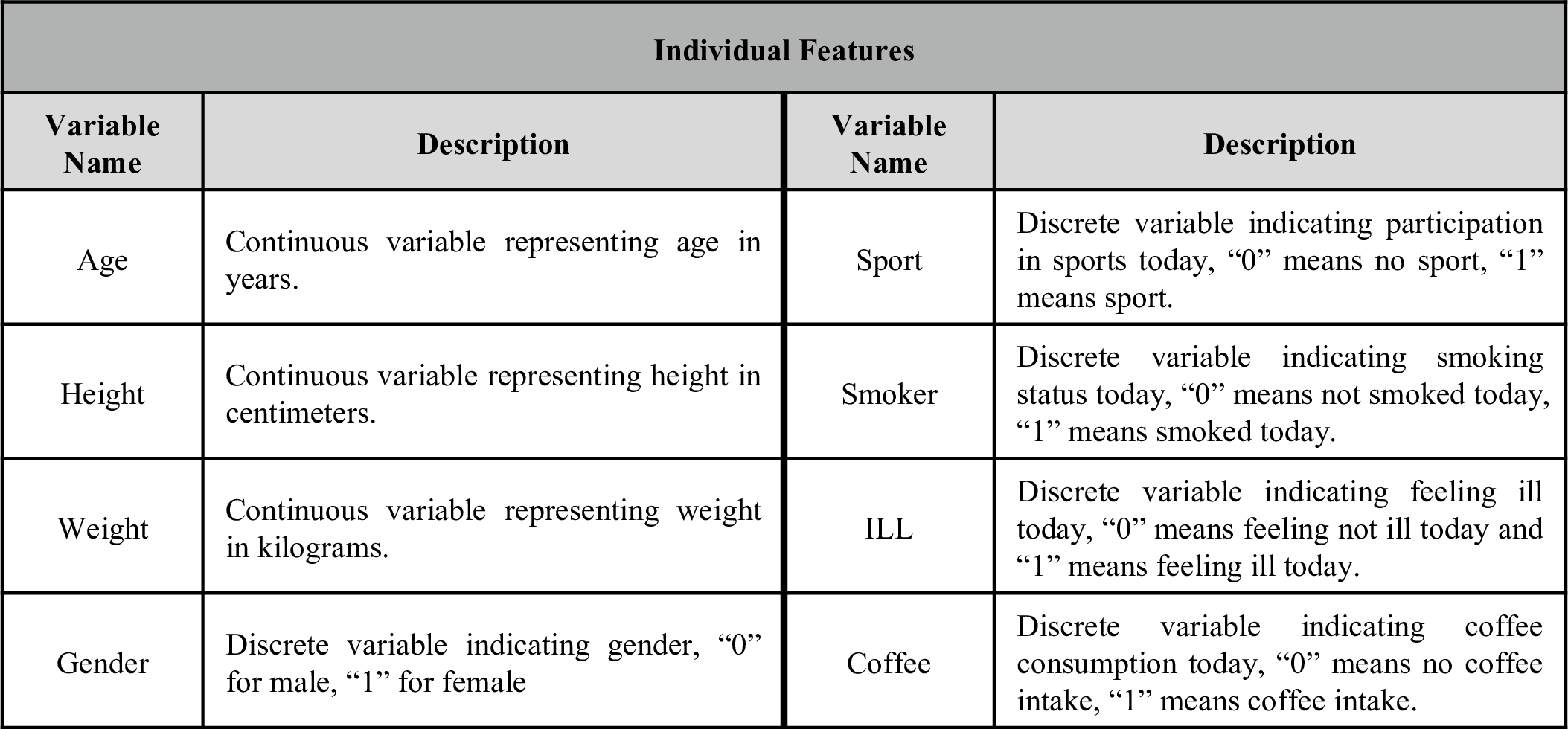

Supplement: S1 Fig — (TIFF) [file pone.0335221.s001.tif]

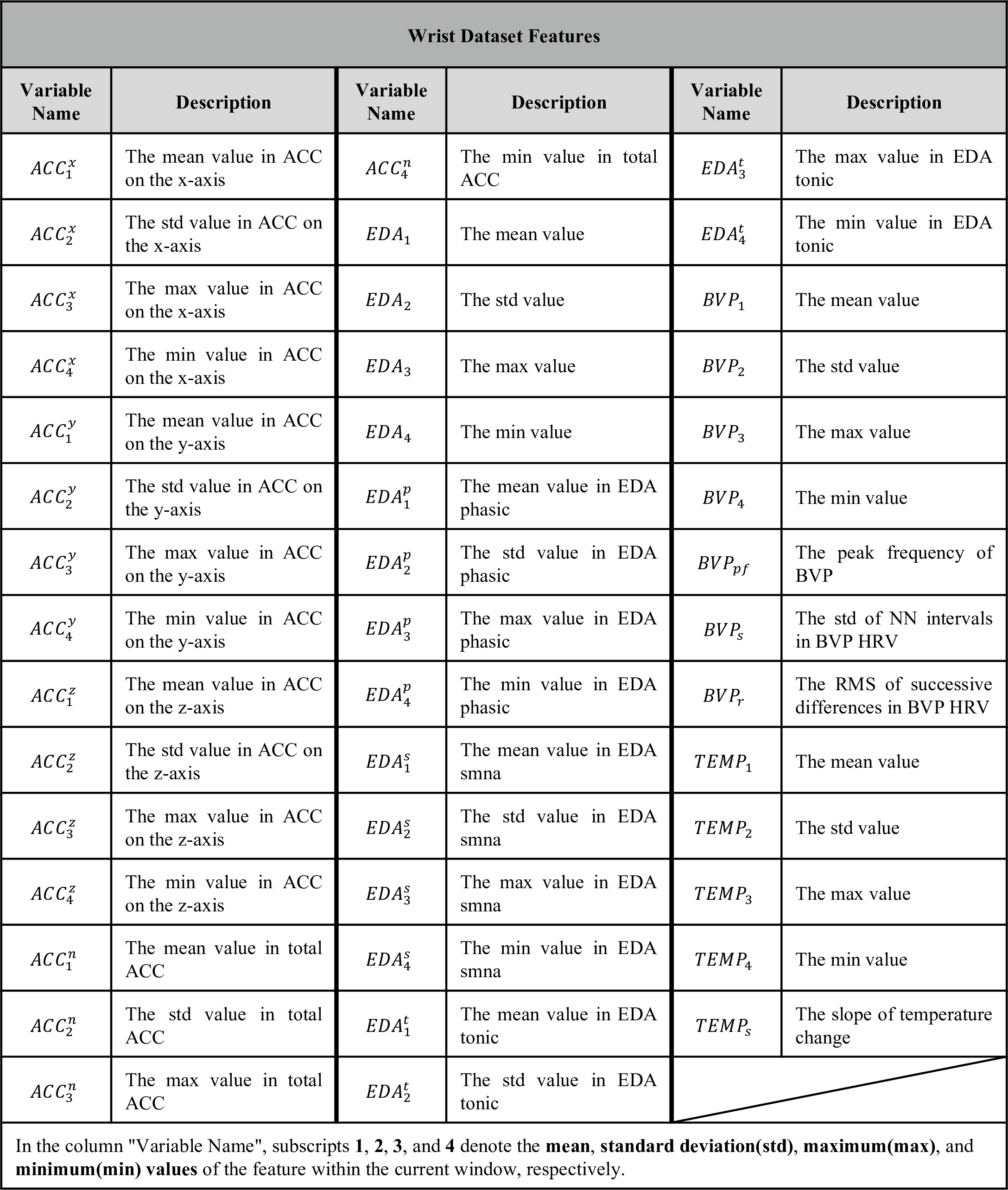

Supplement: S2 Fig — (TIFF) [file pone.0335221.s002.tif]

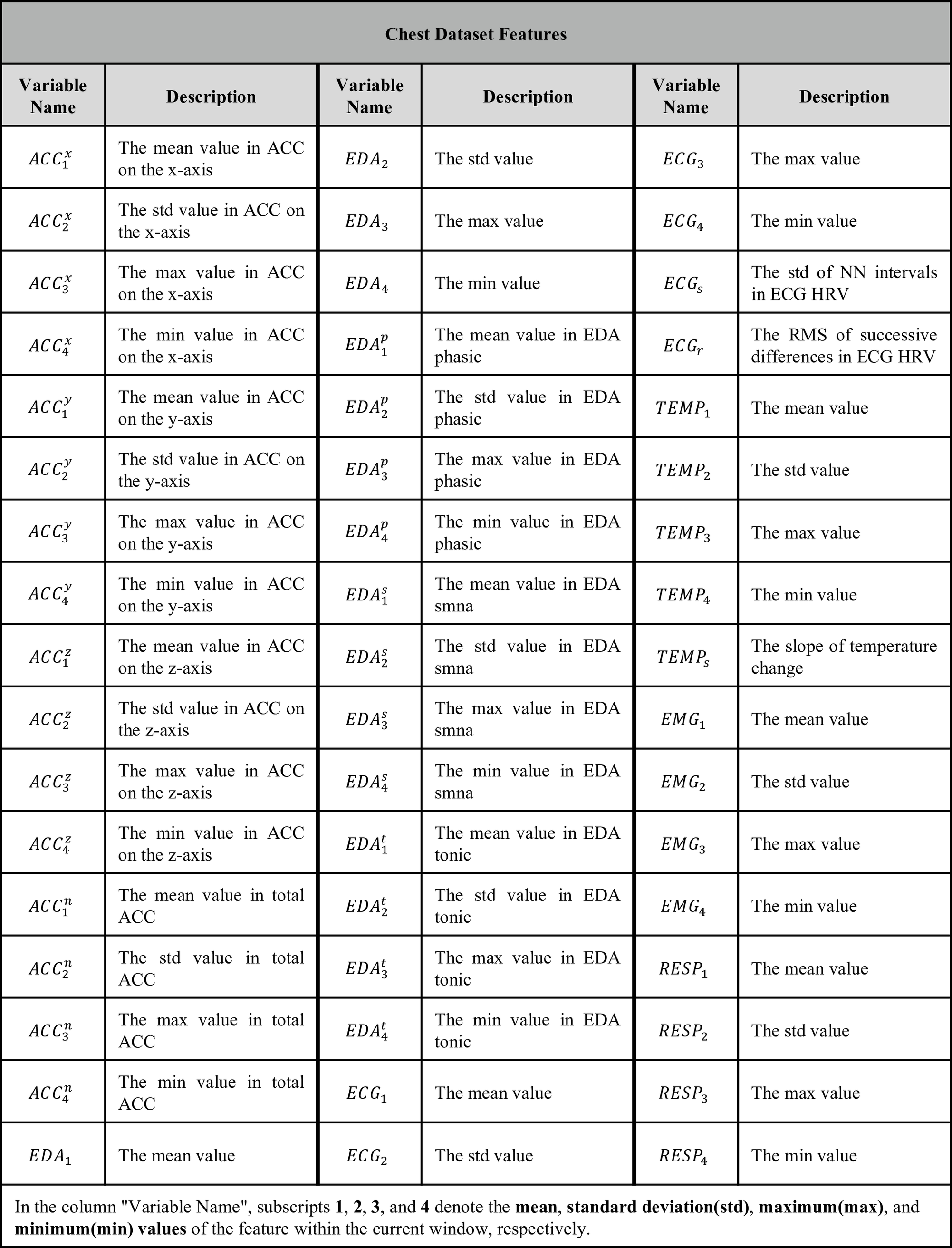

Supplement: S3 Fig — (TIFF) [file pone.0335221.s003.tif]

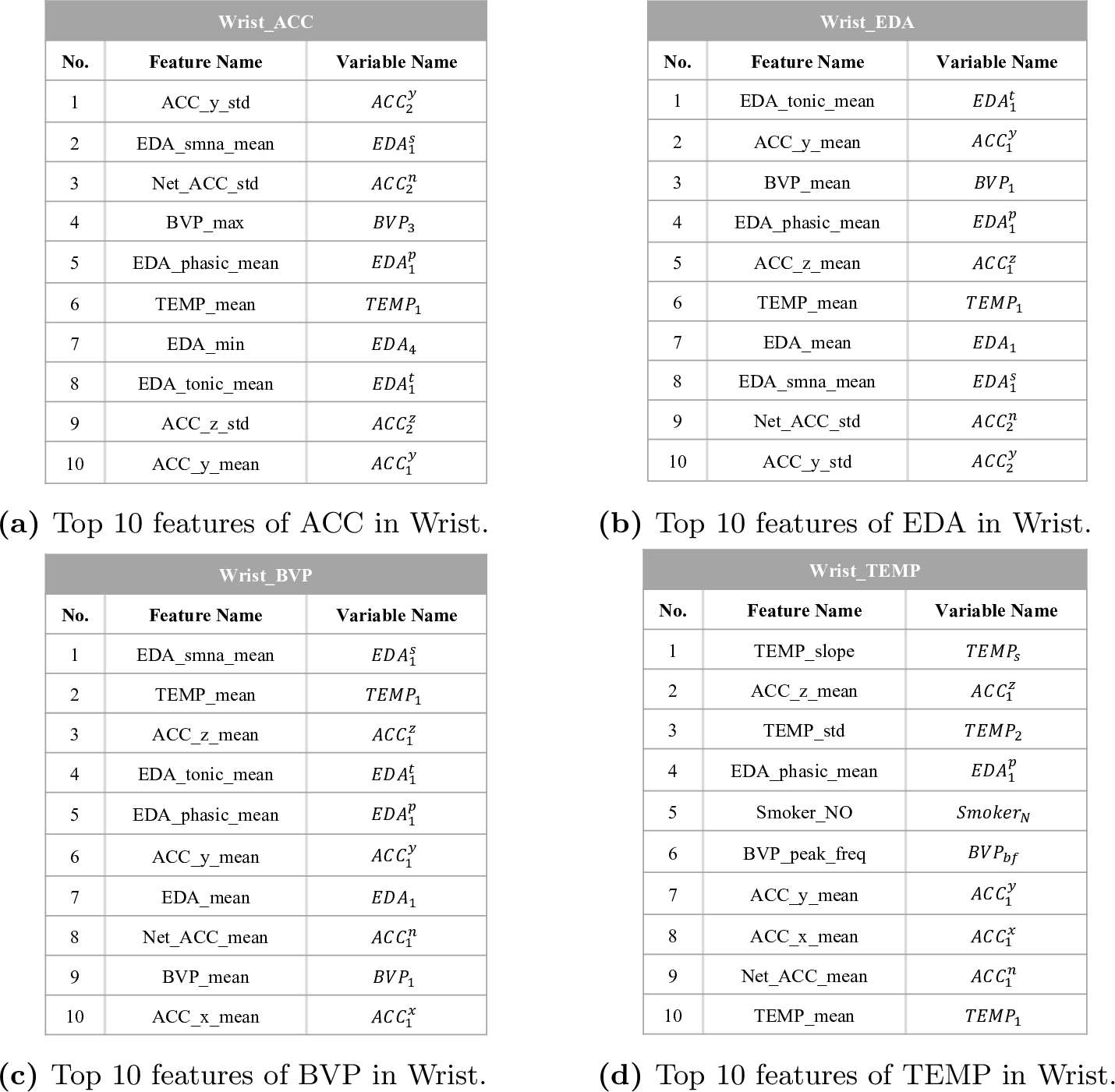

Supplement: S4 Fig — (TIFF) [file pone.0335221.s004.tif]

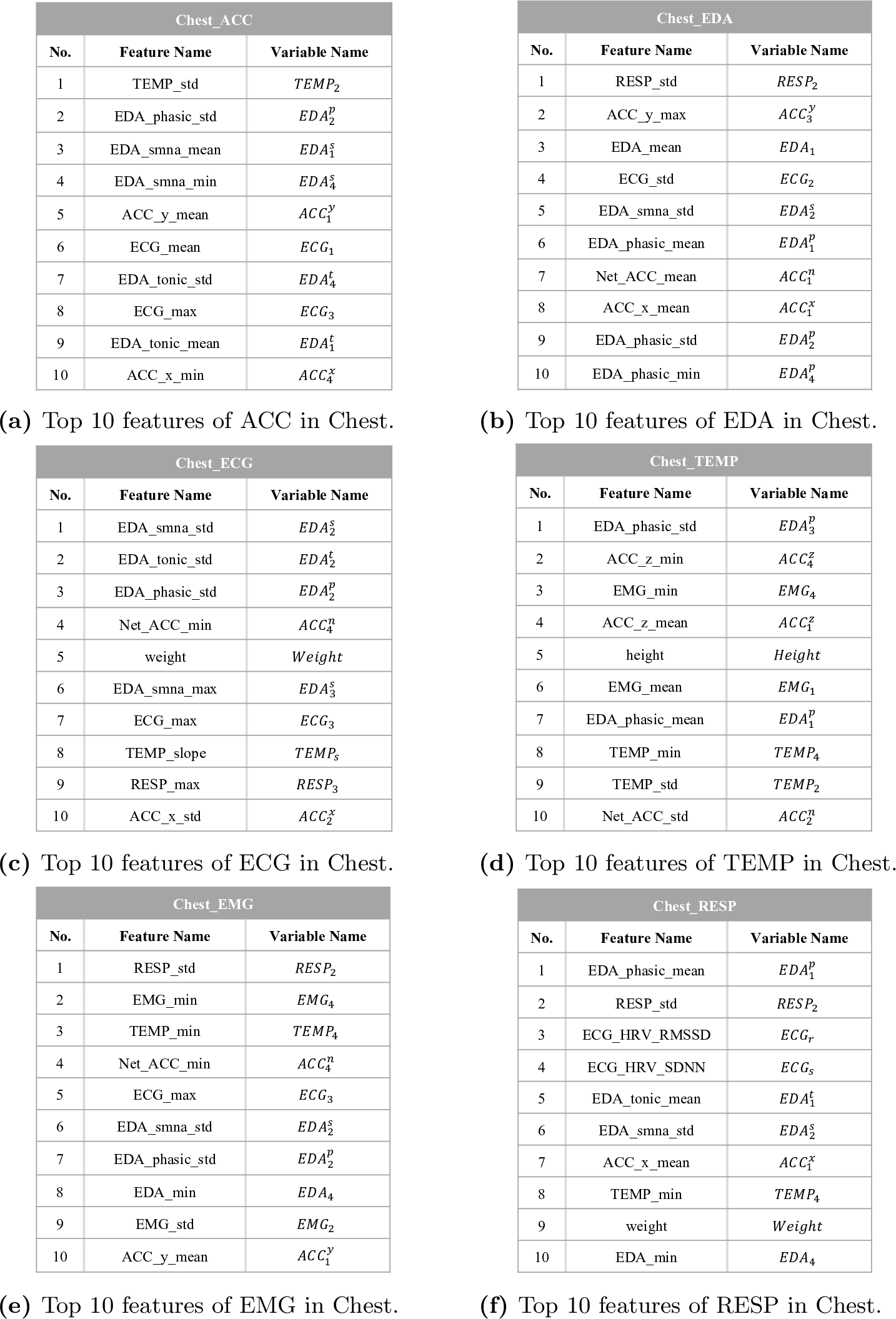

Supplement: S5 Fig — (TIFF) [file pone.0335221.s005.tif]

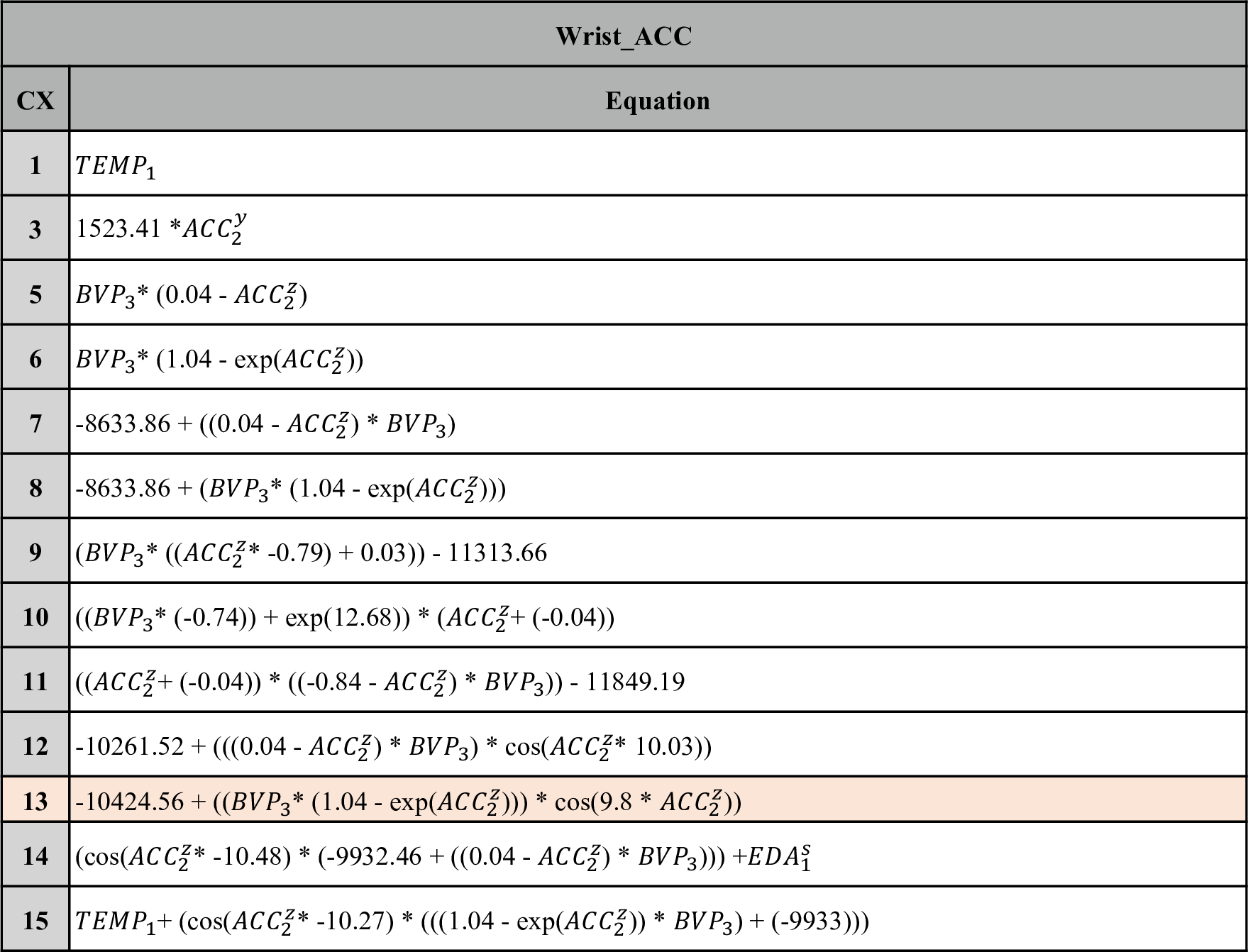

Supplement: S6 Fig — (TIFF) [file pone.0335221.s006.tif]

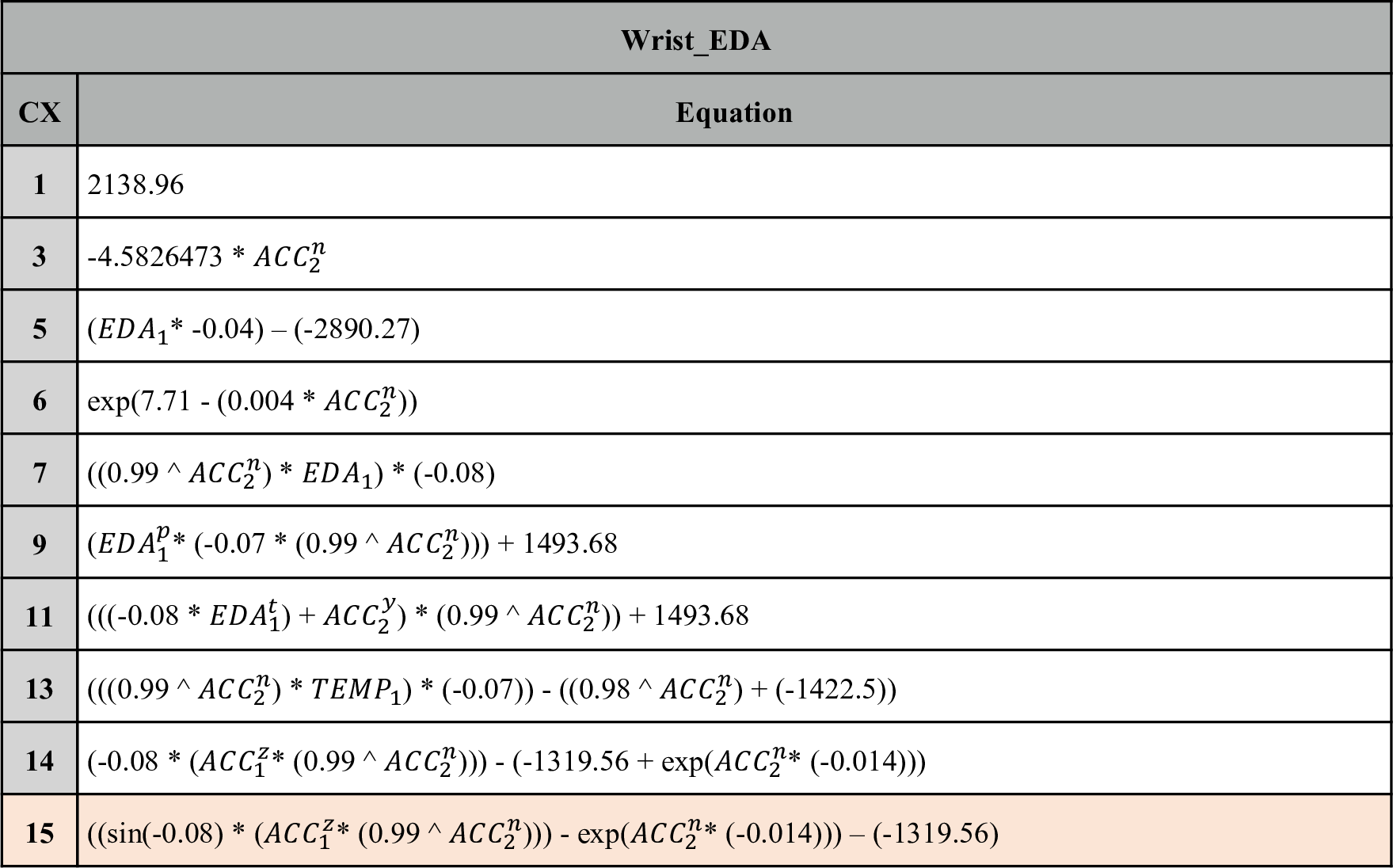

Supplement: S7 Fig — (TIFF) [file pone.0335221.s007.tif]

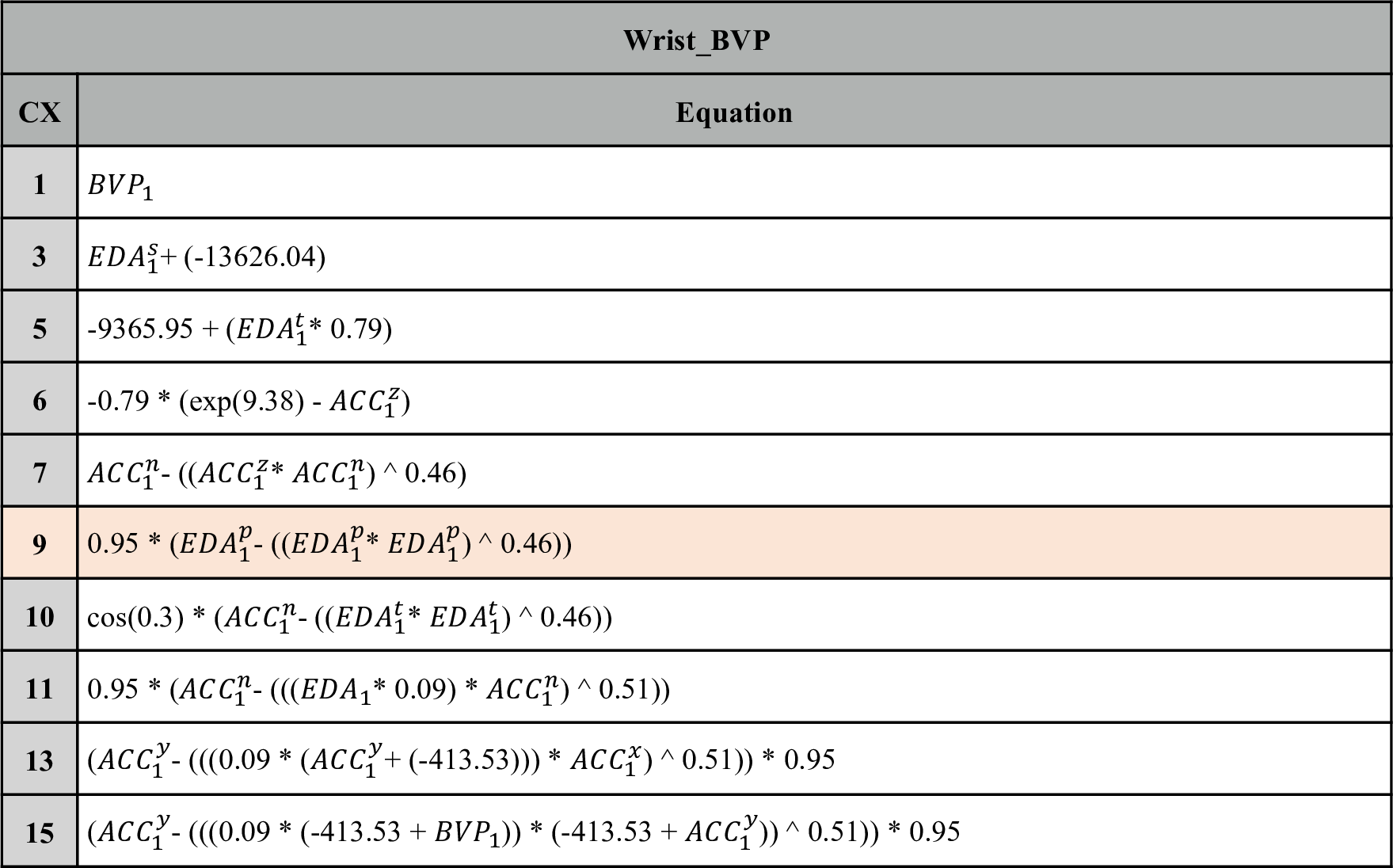

Supplement: S8 Fig — (TIFF) [file pone.0335221.s008.tif]

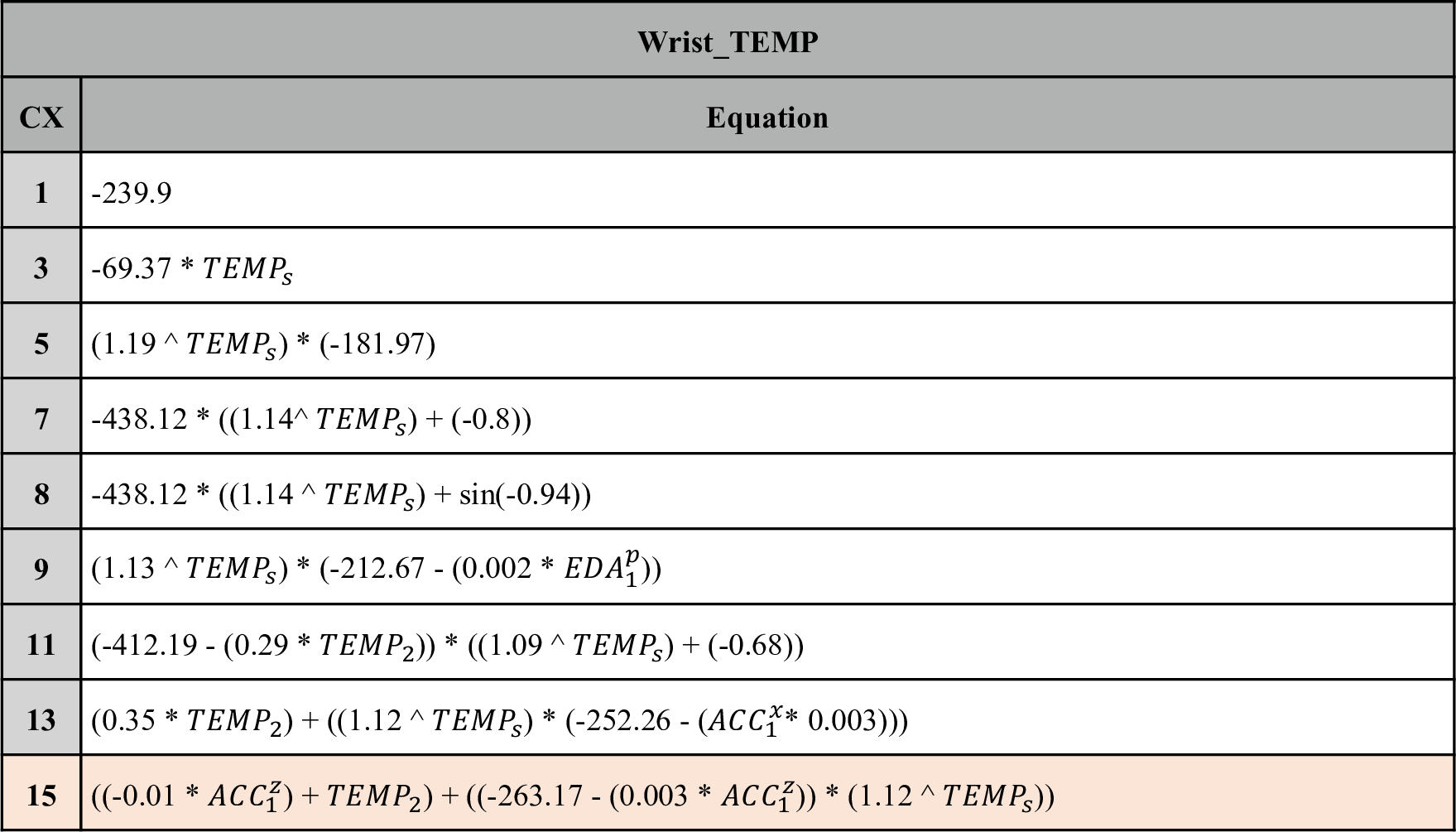

Supplement: S9 Fig — (TIFF) [file pone.0335221.s009.tif]

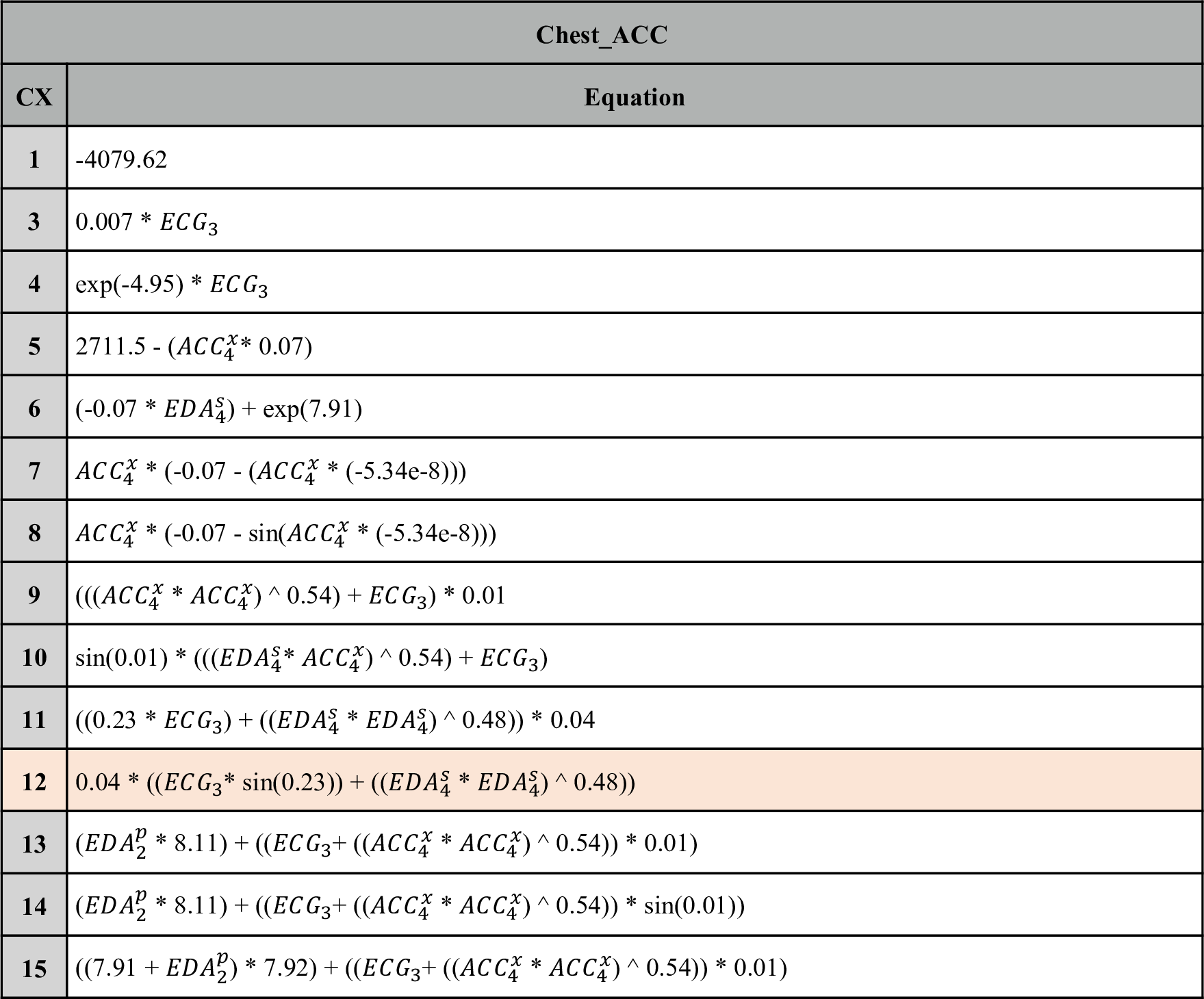

Supplement: S10 Fig — (TIFF) [file pone.0335221.s010.tif]

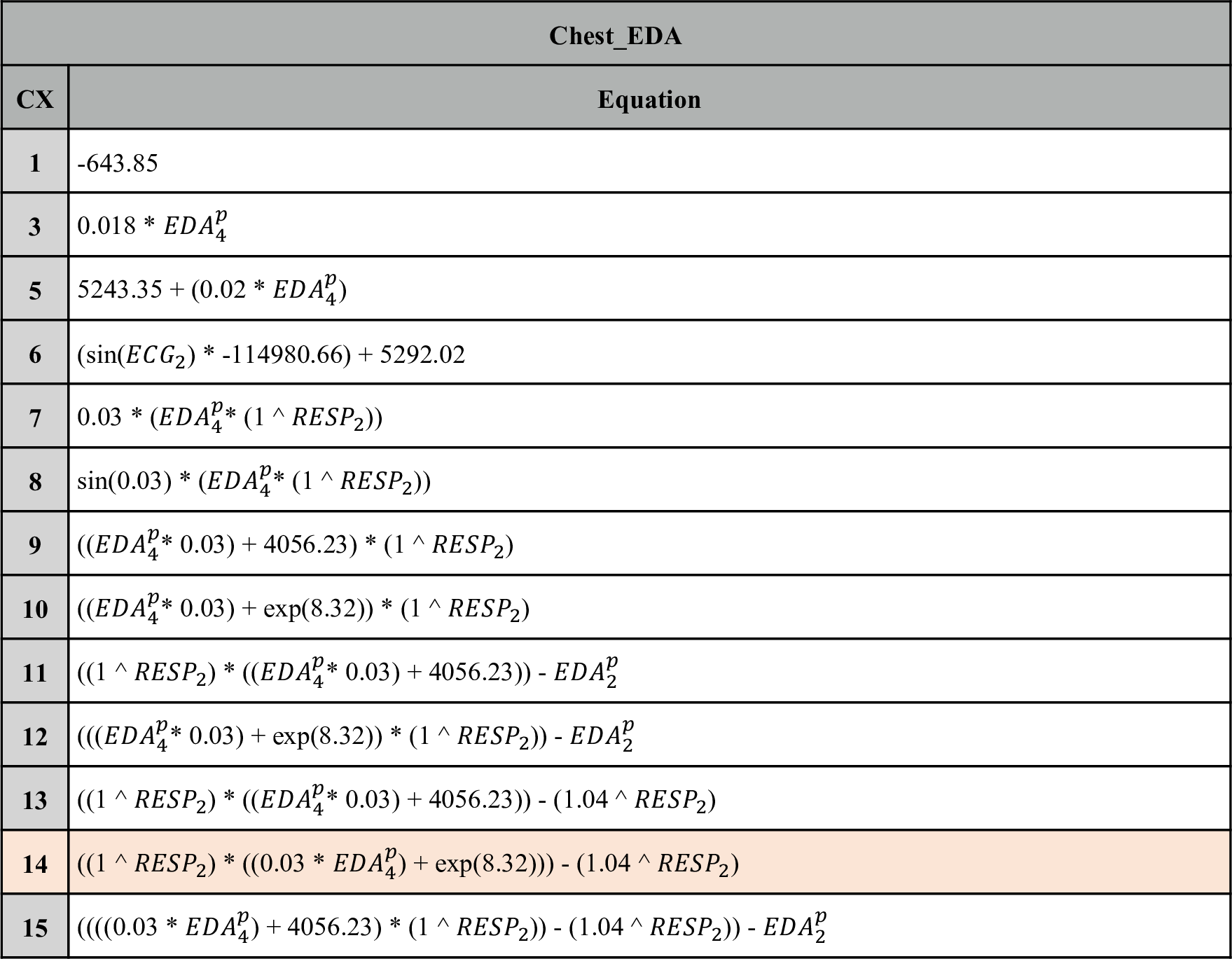

Supplement: S11 Fig — (TIFF) [file pone.0335221.s011.tif]

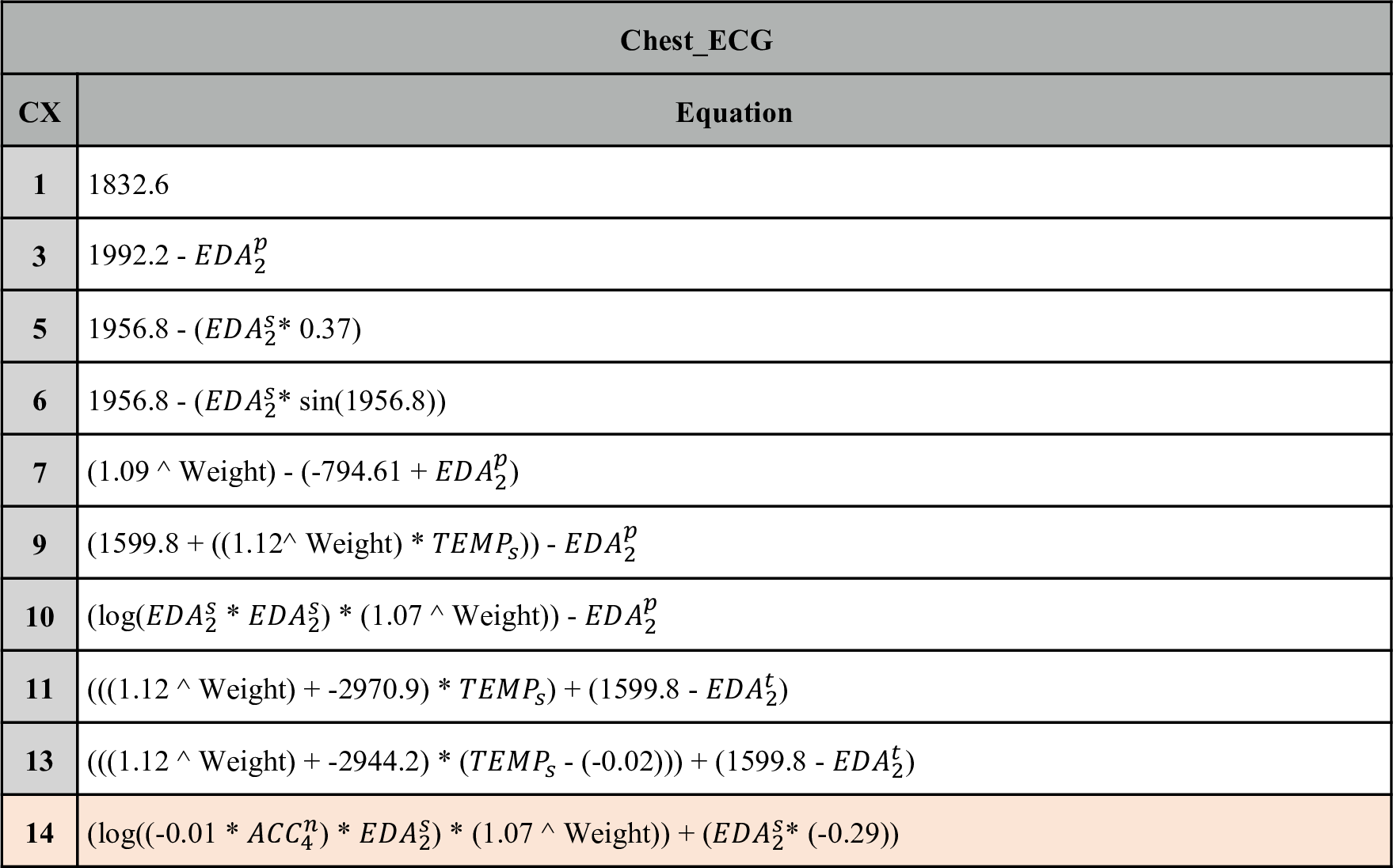

Supplement: S12 Fig — (TIFF) [file pone.0335221.s012.tif]

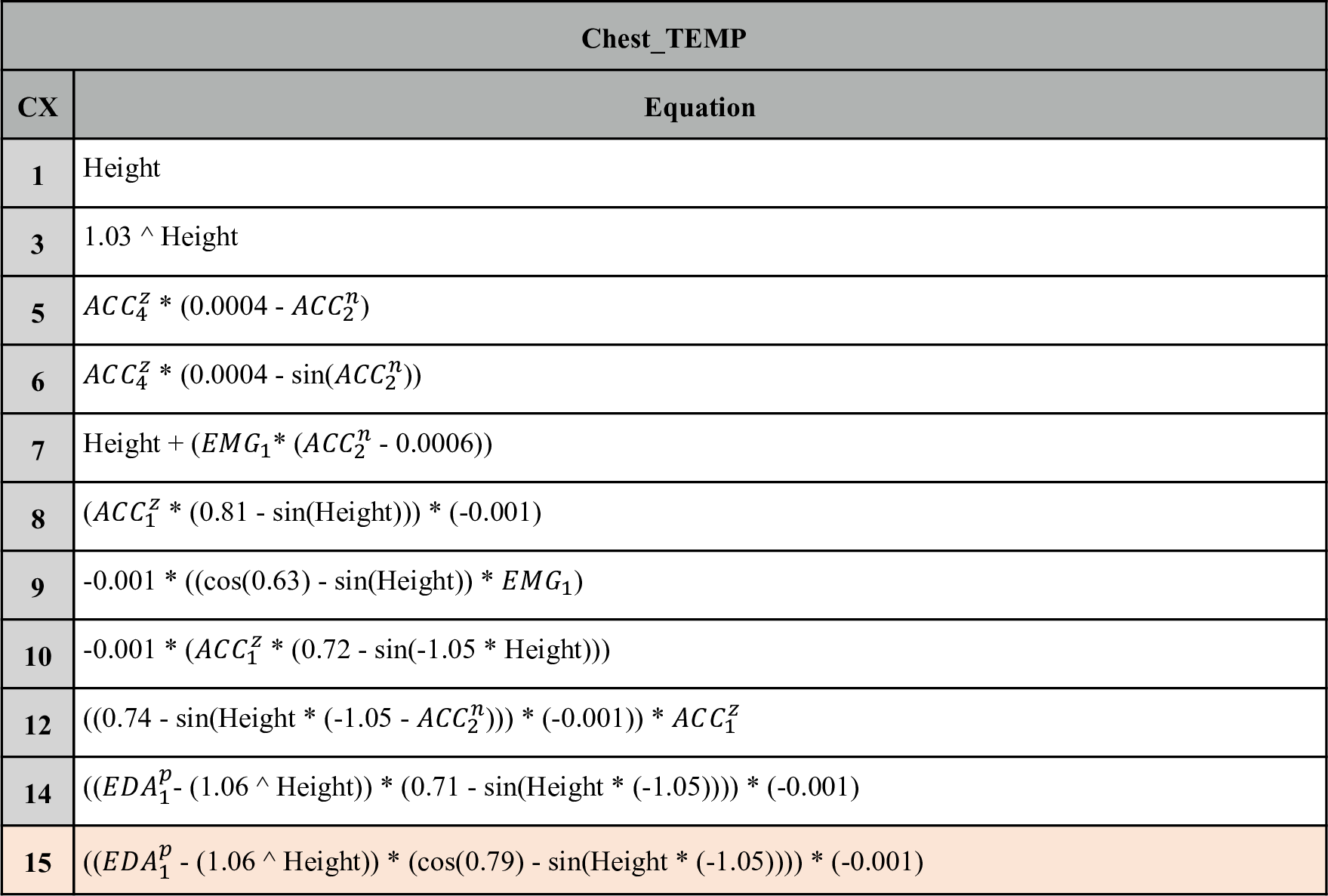

Supplement: S13 Fig — (TIFF) [file pone.0335221.s013.tif]

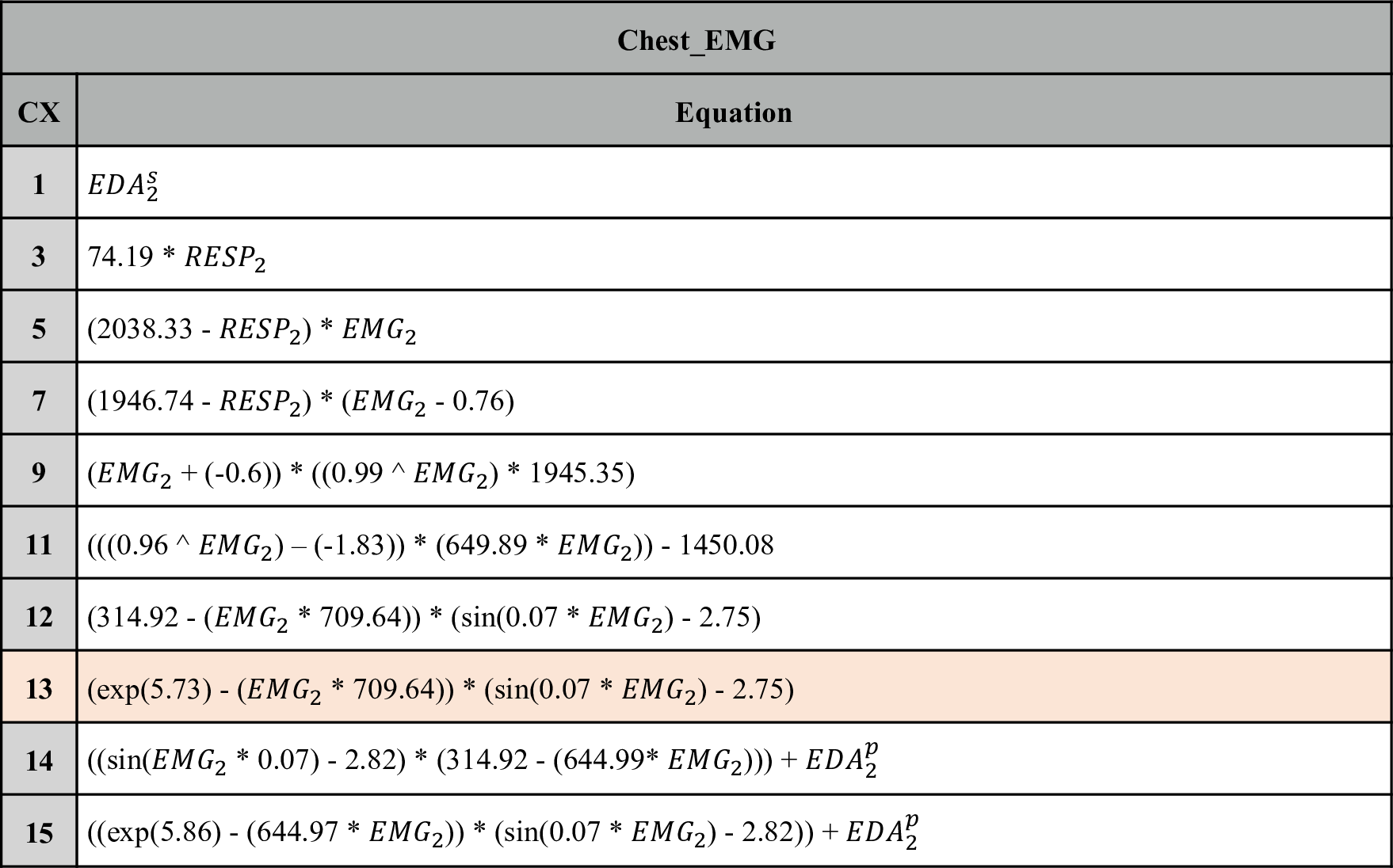

Supplement: S14 Fig — (TIFF) [file pone.0335221.s014.tif]

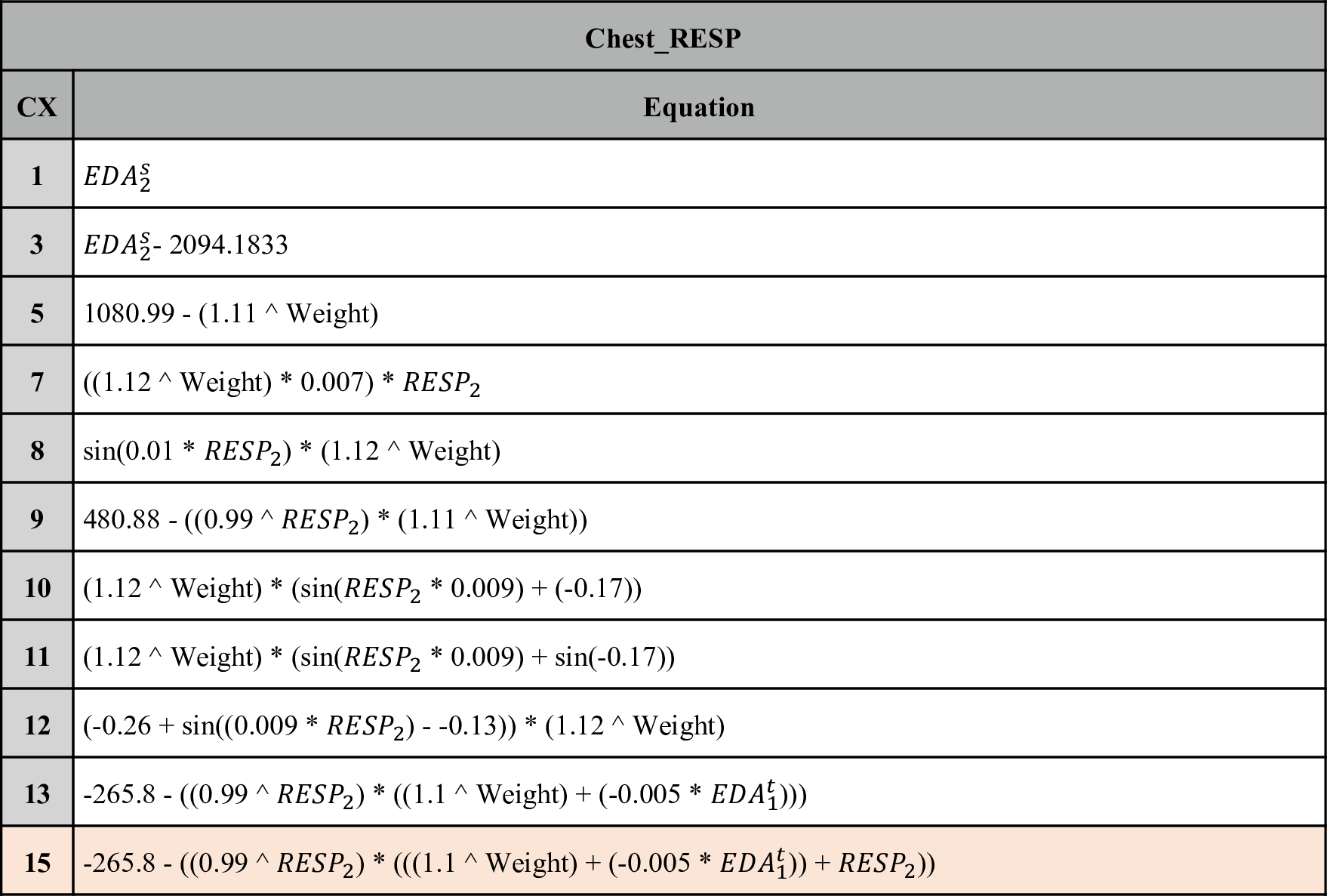

Supplement: S15 Fig — (TIFF) [file pone.0335221.s015.tif]
